# Unraveling Future Trends in Free School Lunch and Nutrition: Global Insights for Indonesia from Bibliometric Approach and Critical Review

**DOI:** 10.3390/nu17172777

**Published:** 2025-08-27

**Authors:** Muhammad Naufal Putra Abadi, Ray Wagiu Basrowi, William Ben Gunawan, Mutiara Putri Arasy, Felasiana Nurjihan, Tonny Sundjaya, Dessy Pratiwi, Hardinsyah Hardinsyah, Nurpudji Astuti Taslim, Fahrul Nurkolis

**Affiliations:** 1 Department of Community Nutrition, Faculty of Human Ecology, IPB University, Bogor 16680, West Java, Indonesia; 2 Department of Community Medicine, Faculty of Medicine, Universitas Indonesia, Jakarta 10430, Indonesia; 3 Indonesia Health Development Center (IHDC), Jakarta 12950, Indonesia; 4 Health Collaborative Center (HCC), Jakarta 10320, Indonesia; 5 INTI International University, Nilai 71800, Negeri Sembilan, Malaysia; 6 PPM School of Management (Sekolah Tinggi Manajemen PPM), Menteng, Jakarta 10340, Indonesia; 7 Department of Nutrition Science, Faculty of Medicine, Diponegoro University, Semarang 50275, Central Java, Indonesia; 8 Department of Epidemiology, Faculty of Public Health, Universitas Indonesia, Jakarta 12345, Indonesia; 9 Division of Applied Nutrition, Department of Community Nutrition, Faculty of Human Ecology, IPB University, Bogor 16680, West Java, Indonesia; 10 Division of Clinical Nutrition, Department of Nutrition, Faculty of Medicine, Hasanuddin University, Makassar 90245, South Sulawesi, Indonesia; 11 Institute for Research and Community Service, State Islamic University of Sunan Kalijaga (UIN Sunan Kalijaga), Yogyakarta 55281, Central Java, Indonesia; 12 Master of Basic Medical Science, Faculty of Medicine, Universitas Airlangga, Surabaya 60115, East Java, Indonesia; 13 Medical Research Center of Indonesia, Surabaya 60286, East Java, Indonesia

**Keywords:** free school lunch program, bibliometric analysis, nutrition policy, child nutrition, sustainability, stakeholder collaboration

## Abstract

**Background:** School lunch programs play a crucial role in shaping the nutritional status and academic performance of children, making them a cornerstone of public health initiatives worldwide. **Objective:** To elucidate emerging trends and propose a comprehensive framework for free school lunch as a nutrition policy through a combined bibliometric approach and critical review. **Methods:** A bibliometric analysis was performed to identify key thematic areas, influential research, and knowledge gaps from global literature databases, followed by a critical review synthesizing insights on nutritional adequacy, socio-cultural considerations, policy effectiveness, and innovative practices in free school meal programs. **Results:** The analysis revealed an increasing focus on sustainability, food waste management, and integration of nutrition education within school curricula, alongside notable disparities in implementation and accessibility, particularly in low-income regions. **Conclusions:** We propose a future-oriented framework emphasizing stakeholder collaboration, culturally adaptive meal designs, and utilization of technology for personalized nutrition strategies, contributing to the optimization of school lunch programs and advancement of sustainable development goals, particularly Zero Hunger and Quality Education.

## 1. Introduction

In 2024, an estimated 150 million children under the age of five were affected by stunting, a condition characterized by impaired growth and development due to chronic malnutrition [1,2]. Indonesia has the second-highest number of severely stunted children globally, with approximately one in five children under five affected [3]. According to the 2023 Indonesia Health Survey, 12.9% of children aged 0–23 months were moderately stunted, and 5.4% were severely stunted [4,5]. These conditions are more prevalent among low-income, rural, and marginalized populations. Stunting is a long-term nutritional issue influenced by multiple interrelated factors such as household economic status, maternal education, access to health services, and adequate sanitation [6]. Furthermore, the effects of early-life malnutrition can persist throughout an individual’s life and even extend to the next generation [7].

To address childhood malnutrition and food insecurity, the President of the Republic of Indonesia, Prabowo Subianto, has introduced a nationwide free school meal initiative known as *Makan Bergizi Gratis* (MBG). This program aims to provide nutritious meals to students across the country, supported by a proposed budget of IDR 71 trillion in 2025. In its initial phase, the MBG program will prioritize students from elementary to senior high schools who belong to the bottom two income quintiles and live in Indonesia’s most disadvantaged “3T” districts (Tertinggal, Terdepan, Terluar). The Prabowo–Gibran transition team has stated that the program’s target groups, funding allocation, and implementation framework will continue to be refined and expanded to help reduce stunting nationwide.

School lunch programs around the world have evolved not only to improve children’s nutritional status, but also to reduce inequality, support local agriculture, and combat hunger [8,9,10]. These multidimensional benefits are also directly connected to the Sustainable Development Goals (SDGs), particularly SDG 2 (Zero Hunger), SDG 3 (Good Health and Well-being), and SDG 4 (Quality Education). Positioning Indonesia’s MBG program within the broader SDG framework offers a future-oriented perspective that can guide its sustainable implementation. In many countries, these programs have successfully achieved such outcomes through clearly defined goals, appropriate beneficiary targeting, and sustainable funding mechanisms. Countries across the U.S., UK, Europe, Africa, and Asia have implemented structured, well-regulated programs that demonstrate the importance of strong policy direction, cross-sector collaboration, and robust nutritional standards [11]. Meanwhile, Indonesia’s current MBG program still lacks a written regulation and a clearly defined national objective. To achieve similar multidimensional benefits, Indonesia must develop a coherent policy framework that outlines the program’s purpose, target groups, and funding responsibilities. This paper reviews international school lunch models to inform the development of an effective, equitable, and sustainable approach for Indonesia. The novelty of this review lies in its comprehensive bibliometric analysis combined with a critical synthesis of international practices, enabling the identification of emerging trends, research gaps, and strategic insights specifically tailored to enhance the effectiveness and sustainability of Indonesia’s free school lunch program (MBG).

## 2. Methods

This study employs a bibliometric approach to quantitatively analyze academic literature on school lunch programs. Bibliometric analysis allows for an objective assessment of research trends, patterns, and influential publications, making it an ideal method for mapping the intellectual landscape of youth entrepreneurship [12]. This approach provides a comprehensive overview of the field by evaluating metrics such as citation counts, keyword co-occurrence, and co-authorship networks, thus uncovering the research domain’s intellectual structure and identifying key themes and developments.

To ensure relevance and rigor, criteria were established for selecting studies specifically related to school lunch programs. In January 2025, a search was conducted in the Scopus database using the keywords (“free school lunch*” OR “free school lunch program*” OR “school lunch program*” OR “free school meal*” OR “free school meal program*” OR “school meal program*” OR “national school lunch program*”). This search resulted in 293 articles identified. This search strategy aimed to capture a wide array of research, encompassing foundational studies and recent advancements, allowing for a well-rounded analysis of prevailing issues and trends. Scopus was chosen as the primary database for this bibliometric analysis due to its extensive coverage of peer-reviewed publications across disciplines [13]. Compared to databases like Web of Science, Scopus offers a broader indexing scope and detailed citation data, making it advantageous for identifying research trends and assessing publication impact. Additionally, Scopus facilitates easy data export, allowing for efficient analysis in VOSviewer (version 1.6.18), a tool designed for bibliometric visualization.

The article selection process followed specific criteria to ensure the relevance and quality of the literature included. There was no restriction on publication year, ensuring comprehensive trend capture. This search yielded 293 relevant documents, which were then exported from Scopus into VOSviewer for visualization and analysis. VOSviewer was used to generate maps based on citation networks, co-authorship, and keyword co-occurrence, enabling a clear visualization of the relationships between authors, institutions, and research themes. To ensure academic relevance, a minimum threshold of keywords was set with a minimum of five occurrences. This process allowed for identifying clusters of research focused on similar themes and pinpointing gaps in the literature where further exploration may be beneficial.

Afterward, a literature review on the topics was performed. The previous databases were then filtered into 189 articles based on research title, focus of the study, and the findings of the study. Furthermore, the literature review was enriched with articles and relevant published reports retrieved from Google Scholar using the same keywords. Utilizing the same set of keywords, other inclusion criteria such as: (i) article in English or Indonesian; (ii) accessible full paper; (iii) fully published journals, reports, or book chapters were included, and (iv) note, review, and editorial papers were excluded.

## 3. Results and Discussion

The results will mainly be divided into two main sections. The bibliometric results highlight the trends regarding school lunch program research, and the literature review results specify the purposes, targeting, impacts, and implications of school lunch programs.

### 3.1. Bibliometric Findings

#### 3.1.1. Time Distribution of Research

**Figure 1:**
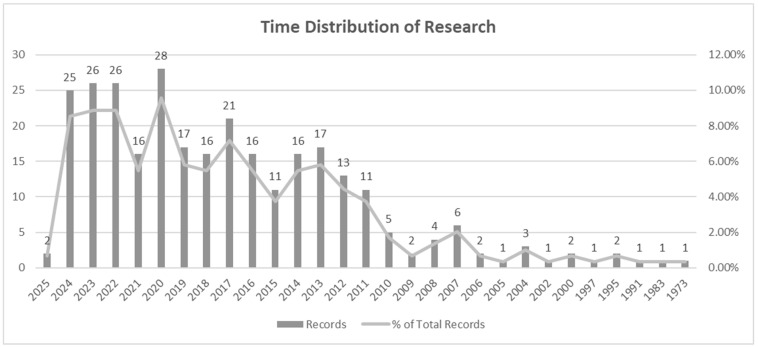
Publications over the year based on the keywords searched.

Figure 1 depicts the temporal distribution of research records spanning from 1973 to 2025, illustrating the fluctuating progression of academic output over this period. Early years, particularly from 1973 to 2005, exhibit minimal activity, with sporadic records that reflect a limited engagement or emerging interest in the subject area. This period is characterized by relatively isolated contributions, with records ranging from one to three annually, indicating nascent scholarly attention.

A notable shift is observed post-2006, where there is a gradual increase in research output, reaching a modest peak of six records in 2007. This growth may signal an expanding awareness or data availability in the field. Between 2008 and 2010, a slight decline was observed, but this was followed by a significant surge starting in 2011, where records consistently climbed, culminating in a marked peak of 28 records in 2020—the highest in the dataset. This peak likely reflects an intense period of academic focus, possibly fueled by contemporary global challenges or advancements in the field.

Subsequent years (2021–2025) demonstrate sustained high output, averaging around 25 records annually, indicative of a stable and mature research interest. The consistency in records during this period suggests that the subject remains a focal point of academic inquiry, potentially driven by ongoing developments, policy implications, or interdisciplinary relevance. The slight variability in these later years could also correspond to shifts in funding priorities, methodological advancements, or external socio-economic factors influencing research production.

Overall, the distribution underscores an evolving trajectory of scholarly engagement, transitioning from an emerging to an established field of study, marked by significant peaks and sustained interest in recent years.

#### 3.1.2. Country Distribution of Research

**Figure 2:**
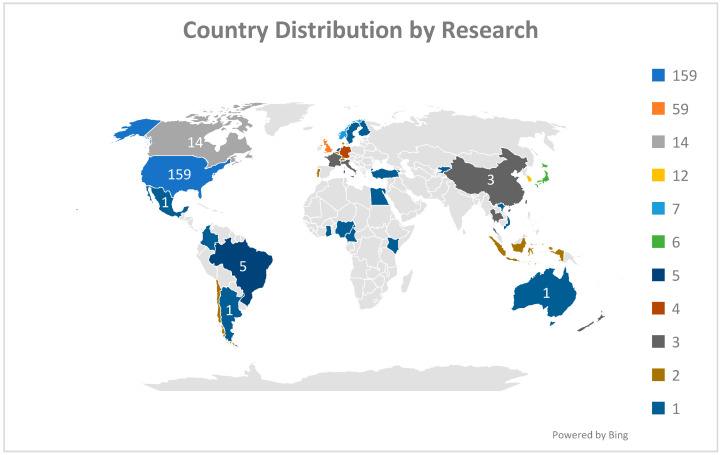
Number of documents published by each country regarding the research topic.

Figure 2 illustrates the global distribution of research outputs, highlighting significant regional disparities in academic contributions. The highest research output is concentrated in the United States (U.S), depicted in dark blue, with 159 records, indicating its leading role in this area of study. Other prominent contributors include United Kingdom (59 records) and select countries, such as Canada and South Korea, which exhibit moderate outputs of 14 and 12 records. In Asia, Japan emerges as notable contributors with 6 records, reflecting growing regional interest. Meanwhile, Latin American nations such as Argentina and Brazil demonstrate limited outputs, with less than 5 records each, signifying a nascent engagement in this research area. Africa, Central Asia, and parts of the Middle East display minimal or no contributions, underscoring a geographical imbalance in research focus. This suggests potential gaps in knowledge and the need for more inclusive, globally representative studies. Overall, the map reveals clear research hubs in North America, Europe, and parts of Asia, while highlighting the underrepresentation of developing regions. This distribution emphasizes the importance of fostering collaborative international efforts to address the uneven geographical scope of research.

#### 3.1.3. Keywords Clusterization and Trend

The bibliometric visualization (Figure 2) presents a keyword co-occurrence network, illustrating the thematic structure of research related to school nutrition programs and public health. This analysis was conducted using VOSviewer, with a threshold of five occurrences per keyword. The network consists of multiple clusters, each representing distinct but interrelated research themes.

The most prominent keyword in the network is “national school lunch program”, which appears centrally with strong connections to various related terms such as “schools”, “child nutrition”, “diet”, “food insecurity”, and “health promotion”. This suggests that research in this domain heavily focuses on government-backed nutrition programs and their impact on child health and well-being. Another frequently occurring term is “human”, which reflects the study population and emphasizes research related to school-aged children and their dietary patterns. Other dominant keywords, such as “lunch”, “article”, and “child”, indicate that a substantial portion of the literature explores meal programs in school settings and their implications for public health.

The network visualization reveals several distinct clusters. The green cluster (nutrition and dietary studies) includes terms such as “diet”, “vegetable”, “nutrient”, “nutritional value”, and “food preference”. Studies within this group likely explore the nutritional composition of school meals, dietary habits, and the impact of meal programs on nutrient intake. The blue cluster (health and physiological outcomes) is characterized by keywords like “child nutrition”, “health promotion”, “school health service”, and “childhood obesity”, suggesting a focus on the physiological and health-related effects of school meal programs, including obesity prevention and general child well-being. The red cluster (food security and policy research) includes “food insecurity”, “poverty”, “food assistance”, and “social policy food supply”, indicating research that investigates the socioeconomic dimensions of school nutrition programs, particularly their role in addressing food insecurity among children from low-income households. The yellow cluster (school programs and evaluations) is centered around keywords such as “program evaluation”, “school health services”, and “school meal program”, highlighting a focus on assessing the effectiveness and implementation of school-based nutrition interventions.

Several emerging themes are evident in the network. Notably, terms related to COVID-19, pandemics, and food security indicate that recent studies have explored the pandemic’s impact on school nutrition programs. The presence of “epidemiology” and “longitudinal study” suggests an increasing interest in long-term health outcomes associated with school meal interventions. Furthermore, terms like “free school meals”, “parents”, and “policy” indicate a growing research focus on equity and accessibility in school nutrition policies. The food stamp program appears as a distinct node, suggesting that researchers are examining the relationship between broader food assistance programs and school meal initiatives.

The findings underscore the critical role of school nutrition programs in promoting child health and reducing food insecurity. Policymakers can leverage insights from these studies to enhance the effectiveness of school meal programs, ensuring they meet the nutritional needs of children while addressing disparities in access. Additionally, the network suggests that further research is needed on the long-term health outcomes of school nutrition policies, the impact of socioeconomic factors, and the effectiveness of dietary interventions in reducing childhood obesity.

**Figure 3:**
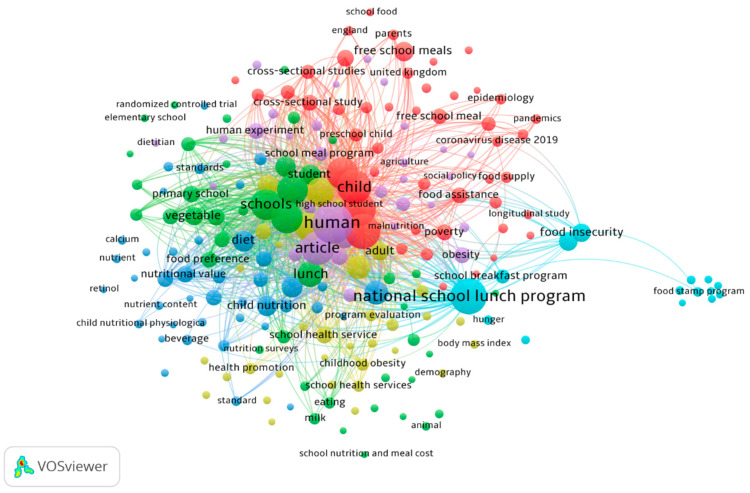
Network visualization of co-occurrences of keywords.

The overlay visualization of keyword co-occurrence presents the evolution of research themes over time in the domain of school nutrition, food security, and public health (Figure 3). The color gradient, ranging from purple (earlier years, approximately 2014) to yellow (more recent studies, approximately 2020), provides insight into the shifting focus of research topics and emerging trends within this field.

#### 3.1.4. Early Research Focus (2014–2016, Purple-Blue Keywords)

In the earlier years, research was heavily concentrated on policy-driven school nutrition programs, as seen in dominant terms such as “national school lunch program”, “food stamp program”, and “school breakfast program”. This suggests that early studies primarily examined the structure and impact of government-supported meal programs on children’s health and food security. Additionally, foundational nutritional concepts, including “nutrient content”, “calcium”, “retinol”, and “child nutritional physiological phenomena”, were key themes, indicating a focus on the nutritional quality of school meals. The presence of “health promotion” and “childhood obesity” suggests that concerns about obesity prevention and overall child well-being were already established research priorities during this period.

#### 3.1.5. Mid-Period Research Themes (2016–2018, Green Keywords)

As research progressed, the focus expanded beyond meal program implementation to the broader implications of school nutrition on children’s dietary behaviors and health outcomes. Keywords such as “food security”, “poverty”, “malnutrition”, and “social policy food supply” reflect an increasing interest in the socioeconomic factors influencing children’s access to adequate nutrition. Terms like “vegetable”, “diet”, “food preference”, and “nutritional value” indicate a growing body of research examining how school meals influence eating behaviors and dietary patterns. Furthermore, studies began incorporating longitudinal studies and program evaluations, suggesting a shift toward assessing the long-term effectiveness of school nutrition policies.

#### 3.1.6. Recent Trends and Emerging Topics (2018–Ongoing, Yellow Keywords)

**Figure 4:**
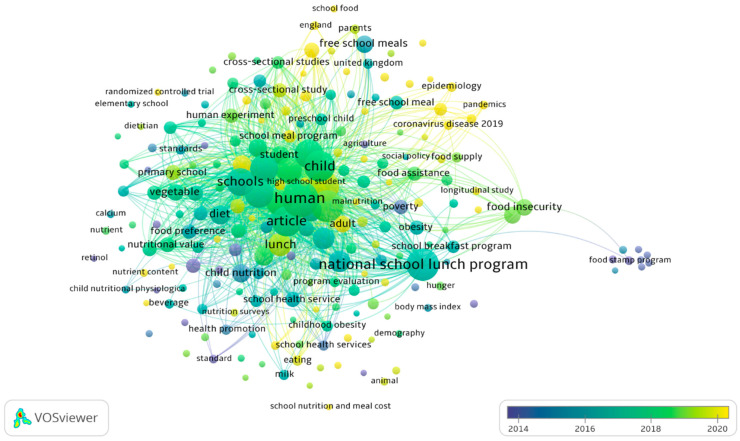
Overlay visualization of co-occurrences of keywords.

In the most recent period, research has increasingly addressed contemporary challenges and new policy directions. Notable recent keywords include “free school meals”, “universal school lunch”, and “food assistance”, highlighting a rising interest in policies aimed at providing equitable meal access for all students (Figure 4). Additionally, “pandemics”, “coronavirus disease 2019”, and “COVID-19” appear as prominent keywords, reflecting the research surge into how the pandemic disrupted school meal programs and exacerbated food insecurity among children. The inclusion of “epidemiology” and “public health” suggests a stronger interdisciplinary approach, linking school nutrition to broader health crises. Moreover, newer research trends include “parental influence” and “behavioral studies”, emphasizing the role of family dynamics in shaping children’s food choices.

Moving forward, future research may increasingly examine the long-term effectiveness of universal free meal programs, the intersection of school nutrition and mental health, and the use of technology in improving meal accessibility and nutritional education. The integration of behavioral science, policy evaluation, and public health will likely continue to shape this field.

### 3.2. Literature Review

#### 3.2.1. Existing Guideline for School Lunch Program

The National School Lunch Program (NSLP) is a federally assisted meal program in the United States that provides free or low-cost lunches to students in public and nonprofit private schools, as well as residential child care institutions. Established in 1946 under the Richard B. Russell National School Lunch Act, it initially focused on preventing undernutrition with calorie- and protein-rich meals. Over time, concerns about poor nutrition led to reforms, including the Child Nutrition Act of 1966, the School Meals for Healthy Kids Initiative in 1995, and the Healthy, Hunger-Free Kids Act (HHFKA) of 2010, which strengthened nutrition standards by increasing fruits, vegetables, and whole grains while reducing unhealthy fats and sodium. In 2024, the United States Department of Agriculture (USDA) introduced updated guidelines lowering sugar and sodium levels further. As part of NSLP, several initiatives like Farm to School, Smart Snacks in Schools, and the Community Eligibility Provision (CEP) help improve child nutrition and expand access to healthy meals [14].

In the United Kingdom (UK), Free School Meals (FSM) aim to improve food access for children from low-income families who meet specific criteria, such as receiving government benefits or earning below a set income [15,16]. FSM applies to students aged seven and above, while children aged four to seven receive meals through the Universal Infant Free School Meal (UIFSM) policy, regardless of income. UIFSM began in 2014 in England and in 2015 in Scotland [17]. To reduce gaps in meal access during holidays, the UK introduced measures like food parcels and vouchers, though support was sometimes absent [18]. In 2018, the government launched the Holiday Activities and Food (HAF) Programme for children from low-income families, offering meals and enrichment activities. It was expanded nationwide in 2021 with GBP 220 million in funding for local authorities [19,20].

FSM policies vary widely across European countries, reflecting different national priorities and welfare models. Finland and Sweden stand out for providing universal free meals to all students, including those in high school and vocational programs. In contrast, countries like Germany and Spain implement targeted approaches, offering subsidized or reimbursed meals specifically for students from low-income families. For instance, in Germany, eligible families can apply for education and participation benefits that cover school lunch costs. In France and Italy, access to school meals depends on regional funding mechanisms, resulting in variation across localities. In larger French municipalities, for example, meal prices are adjusted according to household income [20]. Meanwhile, Denmark and Norway do not have national school meal programs, and students typically bring packed lunches from home. To address growing concerns about children’s dietary habits, Norway introduced national guidelines in 2015 aimed at improving the nutritional quality of these home-prepared meals [21,22].

In Africa, school meal programs have evolved from donor-dependent food aid into nationally led initiatives promoting long-term sustainability. Beginning in the early 2000s, the transition to Home-Grown School Feeding (HGSF) marked a shift from intermittent relief to structured support for domestic food systems, involving local governance and smallholder farmers [23]. Unlike earlier models, HGSF sources food locally, creating stable markets for rural producers and fostering intersectoral collaboration among education, agriculture, health, and social protection sectors. These programs address food insecurity, improve school attendance and completion rates, and advance child nutrition, economic development, and gender equity. Meals are prepared in schools using locally sourced ingredients, reinforcing short supply chains and strengthening school–community ties. While some programs still rely on donor aid in early stages or emergencies, HGSF increasingly emphasizes national ownership and sustainable resource use [23].

South Korea’s school meal program began in 1953 as a relief initiative and evolved into a universal welfare service by the 2010s. It is defined by universal access, on-site meal preparation, and integrated nutrition education. Meals follow Korean Dietary Reference Intakes (DRIs) and use local ingredients through a farm-to-school system, supported by trained dietitians. The program also strengthens local economies and is overseen by a multi-level governance system involving school staff, regional offices, and national institutions [24]. A key development is the Eco-Friendly Free School Meal Program (FSMP), which was introduced to promote equity and reduce stigmatization. Although FSMP successfully expanded nationwide and improved access to nutrition, it created fiscal trade-offs. The use of school discretionary funds (SDFs) to support FSMP often reduced budgets for areas like physical education, contributing to declines in student fitness. This highlights the need for balanced planning to maintain overall educational quality while achieving health and equity goals [25].

Japan’s school lunch program has operated since 1889, formalized under the School Meals Act of 1954. Regulated by the Ministry of Education, Culture, Sports, Science and Technology (MEXT), the program uses nutrient-based standards to deliver 33–50% of students’ daily nutrient needs based on age-specific DRIs. Japan’s program maintains consistent quality and national coverage, promoting health and growth for all school-aged children [24].

Taiwan’s school lunch initiative began in 1951, becoming a formal policy through the School Health Act in 2002 and revised in 2013 to incorporate nutritional standards. Overseen by the Ministry of Education, the program combines food- and nutrient-based guidelines based on the 2011 Taiwanese DRIs, targeting about one-third of daily nutrient requirements. Both countries not only focus on macronutrients, but they also focus on micronutrients such as calcium, iron, and multiple vitamins, though sodium and fat are not yet regulated [24].

China’s Nutrition Improvement Program for Rural Compulsory Education Students (NIPRCES), launched in 2011, is a centrally funded initiative to address malnutrition in rural areas. The program is implemented by local governments, offering flexibility in meal preparation—either through in-school kitchens or catering services. Locally sourced food supports regional agriculture and aligns with cultural dietary habits [26].

Kyrgyzstan’s school meal program began in 2006, offering free meals to public school students in Grades 1–4. The program aims to improve child nutrition and attendance, particularly in regions with high poverty. Meals are simple and low-cost, typically consisting of bread and milk, served 170 days a year. The program is guided by a national legal framework and implemented in nearly all regions of the country, but the rural areas still lag behind despite international assistance [27].

Brazil’s national school feeding program, the Programa Nacional de Alimentação Escolar (PNAE), is one of the largest and most structured in the world. Established in 1955 and reformed in 2009, the program provides universal access to nutritious meals for students in public schools, ensuring at least 30% of food is sourced from family farming. The policy framework promotes not only child nutrition but also sustainable agriculture and local economies. Meals are aligned with national dietary guidelines and are planned by trained nutritionists. One key strength is its integrated governance structure involving federal, state, and municipal levels [28,29,30].

#### 3.2.2. Purpose of School Lunch Program

School meal programs worldwide primarily aim to enhance children’s nutrition and overall health (Table 1). In Africa, 88% of school meal initiatives focus on improving nutrition, with a smaller fraction (10%) explicitly addressing obesity through varied and balanced meals [23]. Similarly, China seeks to improve the health and nutrition of students in rural areas by providing nutritious meals and enhancing school cafeterias. While no strict food standards exist, schools are encouraged to incorporate nutrient-rich foods such as meat, eggs, milk, and vegetables, accompanied by educational guides on healthy eating for students, parents, and staff [26]. In Kyrgyzstan, the FSM program is specifically designed to improve nutrition among primary school children, particularly in food-insecure regions [27]. Likewise, in South Korea, the school meal program is structured to support children’s healthy growth and development through high-quality meals [31].

Beyond improving nutrition, school meal programs serve as a platform for dietary education, equipping children with the knowledge and skills to make healthier food choices. In the UK, the School Food Plan integrates nutrition education into the school curriculum alongside providing healthy meals through the FSM program. This initiative aims to instill social and moral values by educating students on cooking and healthy eating habits, promoting long-term well-being [32]. Similarly, Kyrgyzstan’s FSM program is linked to higher school attendance and academic performance, emphasizing the role of proper nutrition in cognitive and educational outcomes [27]. South Korea also recognizes the educational potential of school meal programs, using them as tools to teach students about nutrition and encourage lifelong healthy eating habits [31].

Many school meal programs act as a socioeconomic safety net, ensuring food access for vulnerable children. A study in 41 African countries revealed that 81% of school meal programs serve this function, targeting disadvantaged populations to alleviate food insecurity [23]. In the United States, NSLP policies in California and Maine have helped families save money, reduce meal preparation time, and lower financial stress. Parents in these states reported that NSLP eliminated the stigma associated with applying for meal assistance, allowing all children to benefit from free meals without embarrassment. However, concerns about meal quality and healthfulness remain, indicating room for improvement in balancing accessibility and nutritional value [33].

School meal programs can also serve agricultural objectives by fostering connections between local farmers and educational institutions. In the U.S., the Farm-to-School (FTS) program aims to enhance children’s nutrition by integrating locally sourced foods into school meals, thus promoting healthier diets while supporting local farmers [14]. Similarly, in Africa, 46% of school meal programs incorporate HGSF, which prioritizes sourcing food from smallholder farmers to stimulate local agricultural economies [23]. Brazil’s National School Feeding Program (PNAE) furthers this initiative by mandating that at least 30% of the budget for school meals be allocated to purchasing agricultural products from family farmers, reinforcing both food security and economic sustainability [29,34].

School meal programs are instrumental in addressing food insecurity by ensuring reliable access to nutritious meals for children. In the U.S., the USDA’s Child Nutrition Programs (CNPs)—including the NSLP, School Breakfast Program (SBP), and Summer Food Service Program (SFSP)—are designed to combat food insecurity through structured meal provision [35].

#### 3.2.3. The Price of the Program: Universal vs. Subsidized

Universal programs provide free meals to all students, regardless of socioeconomic background. In the UK, the introduction of UIFSM for children in the first three years of primary school led to a significant increase in participation and nutritional quality. Uptake among previously eligible children increased modestly, while a much larger rise was observed among children who were not previously covered, particularly in areas with high economic inequality [15,17,36]. While this inclusive model helps reduce stigma and encourages higher participation, concerns have been raised about cost-effectiveness, as a substantial portion of government funding ends up supporting children from non-low-income families [37].

Subsidized programs offer free or reduced-price meals based on family income or other eligibility criteria. In the U.S., the USDA’s Child Nutrition Programs (CNPs), which include the NSLP and SBP, are federally funded and designed to support students from low-income households. Children from families earning ≤130% of the federal poverty level receive meals at no cost, while those with incomes between 130 and 185% qualify for reduced-price meals [35]. In China, NIPRCES takes a different approach by channeling funding to rural schools based on enrollment numbers rather than individual student eligibility. This gives schools autonomy to plan and deliver meals while balancing funding responsibilities between central and local governments [26]. Across African countries, funding for school feeding programs often comes from a combination of government sources (45%), international donors (51%), and local communities or private businesses (4%) [23].

#### 3.2.4. Appropriate Targeting for Program Participant (Equality, Demographic, Justice, or Equity)

Appropriate targeting is essential to ensure that free lunch programs effectively reach children most in need. Across many countries, eligibility is typically determined through income thresholds or categorical criteria such as enrollment in other welfare programs. However, these methods often fall short in identifying all vulnerable populations. For instance, in England and Wales, although 31% of children live in poverty, only 18% receive FSM, with undercoverage affecting up to 20% of eligible children due to administrative barriers or lack of awareness [16]. A similar trend is observed in the U.S., where participation in the NSLP is relatively high among categorically eligible children, including those in foster care or from households receiving Supplemental Nutrition Assistance Program (SNAP) benefits. However, participation is notably lower among low-income students who must apply based on income [38,39]. While the CEP, which is a federally funded program that allows eligible school districts, offer a path to expand access by allowing schools with high poverty rates to provide free meals to all students, adoption remains uneven. In the 2018–2019 school year, only half of eligible school districts opted into the program, often depending on state-level administrative support and certification systems [40].

Beyond issues of under-enrollment, a significant number of food-insecure children are completely excluded from programs due to restrictive eligibility rules. In the UK, significant numbers of food-insecure children do not qualify for FSM under current criteria, suggesting that eligibility frameworks may be too narrow to address the full scope of need [15]. Furthermore, children from families with “no recourse to public funds” due to their immigration status are often excluded from FSM despite living in extreme poverty. These exclusions raise important concerns about the equity and inclusiveness of current eligibility structures and show how technical definitions of eligibility can unintentionally reinforce systematic marginalization [41].

Targeting strategies also differ widely across global school meal programs, shaped by national income levels and institutional priorities. In middle-income countries, 17 programs employ a universal approach by providing meals to all children, while 15 use geographical targeting in high-poverty or food-insecure regions. Only four programs adopt individual targeting based on specific indicators such as poverty, ethnicity, or malnutrition [42]. Broader data from 85 countries show a clear gradient in school meal coverage according to national wealth: coverage averages 17% in low-income countries and 37% in high-income ones [43].

#### 3.2.5. Revealed Impacts of School Lunch Program on Nutrition and Health

By providing consistent meals, school lunch programs play a crucial role in reducing food insecurity. In Brazil, school meals are vital for lower-income children, many of whom arrive at school without breakfast [44]. In the U.S., NSLP increased the number of free meals served [45] and reduced food insecurity by 2.3–9% [46,47]. Additionally, NSLP and SBP had a greater positive impact on children from food-insecure households, improving their diet quality 2.5 times more than the general population [48]. While school meals help stabilize children’s food intake, seasonal gaps remain a concern [49]. Research finds that food insecurity worsens in school breaks when NSLP is unavailable, increasing by 5% among participants [39,50,51]. A Norwegian study showed that the FSM trial had no significant impact on meal frequency but may have influenced weekend breakfast habits [52]. In England, a universal FSM pilot did not significantly reduce food insecurity but decreased stigma and family stress [53]. Overall, while school meals serve as a crucial safety net for food-insecure children, challenges such as seasonal availability and varying program effectiveness remain.

Beyond addressing food insecurity, school meals shape nutritional status and long-term health, but their impact varies across different settings, particularly regarding obesity. In the U.S., studies show mixed results. The NSLP was linked to higher BMI percentiles in girls [54], and some research suggests that NSLP and SBP participation increased the short-term risk of overweight among low-income students [55] and contributed to higher BMI and obesity risk [56]. However, broader analyses found no significant long-term effects after policy adjustments [57]. Additionally, a study by Localio et al. found that participation in the NSLP, particularly the free lunch program, reduced obesity prevalence by 0.6% and increased normal weight prevalence by 0.58% [58].

Some school meal policies have shown positive impacts in other countries. In England, UIFSMs lowered obesity rates, particularly in younger children [59]. In South Korea, discontinuing free school meals led to weight loss in underweight girls, while reinstatement helped overweight students lose weight [60]. Additionally, a study in Taiwan found that school-prepared meals significantly reduced obesity risk compared to restaurant-prepared meals, which contained more fat and calories [61].

The impact of school meal programs extends beyond physical health to academic performance and cognitive benefits. Better nutrition directly influences students’ academic performance, attendance, and concentration. In Kyrgyzstan, long-term free meal participation reduced absenteeism by 10% [27]. Certain programs have shown improvements in academic outcomes, though the effects vary. In the U.S., increased lunch calories on test days temporarily improved 5th-grade scores [62], while healthier meals under HHFKA led to improved test scores, particularly for low-income students [63]. In Argentina, school meal programs improved language scores but had limited effects on other subjects [64]. In England, FSM students enrolled in certain subjects, including English, math, science, either geography or history, and a foreign language, when barriers were removed [65]. However, South Korea’s school meal education programs showed weaknesses, with low scores in nutrition education [31].

Dietary quality also improves with school meals, as they contribute significantly to children’s daily nutrient intake. In the U.S., based on NHANES (2007–2012), NSLP and SBP provided 47% of students’ daily energy intake, 58% of their fruit, 41% of their vegetables, 52% of their grains, and the majority of their milk consumption [66]. Stronger nutritional guidelines further enhance these benefits. For example, after being implemented, HHFKA meal standards led to healthier options, increasing whole grains, fruits, and vegetables compared to previous standards [67]. In the U.S., additional programs like an FTS improved dietary quality by increasing fiber, protein, and whole grains while lowering sugar, fat, and sodium intake [14]. Furthermore, the UIFSM policy in the UK has led to increased uptake of school meals and a reduction in the consumption of unhealthy foods such as crisps, with more significant dietary improvements observed among low-income children [68,69]. Although UIFSM did not significantly increase fruit and vegetable intake, it contributed to reducing dietary disparities and promoted greater consumption of minimally processed foods among disadvantaged groups [69].

School meals also shape long-term eating behaviors. In the U.S., the FTS program encouraged parents to replicate school meals at home [14], while UIFSM in the UK helped low-income families transition to minimally processed foods [69]. Between 1999 and 2012, Healthy Eating Index-2010 (HEI-2010) scores, which measure diet quality, improved significantly [70]. Further HHFKA updates increased HEI scores, especially for low-income students [71,72]. These programs led to higher whole grain, fruit, and dairy intake while reducing empty calorie consumption [73,74]. Norwegian FSM trial participants had higher Healthy Food Scores, with low-income children benefiting the most [22]. However, there are concerns regarding added sugar intake. NSLP participants in the U.S. consumed more added sugar, particularly Latino students [75], although they had better overall lunch quality than non-participants [76]. Programs like “Chefs Move to Schools” and Offer Versus Serve (OVS) increased fruit, vegetable, and whole grain intake [77], while salad bars in Title I schools led to greater variety but smaller portion sizes [78]. In Europe, FSM reduced family dependence on home-prepared meals, but parental encouragement remained crucial for fruit and vegetable intake [21]. NSLP participation also increased milk and vegetable consumption while reducing sugary beverages [79].

Despite these benefits, school meal programs also present unintended challenges. In the U.S., NSLP participation was linked to higher odds of cigarette use among youth, particularly White and Latinx students, highlighting the need for targeted smoking prevention programs [80]. In South Korea, the temporary removal of free school lunches increased student distress, but mental well-being improved when the program was reinstated [60]. However, South Korea’s FSMP also led to a decline in student fitness levels due to reduced school sports funding, affecting even high-income schools [25]. Another concern is excessive sugar in school meals, with over half of U.S. children consuming more sugar than recommended, mainly from flavored milk and sweetened cereals [81].

#### 3.2.6. Key Success Factors

Key drivers of success in school meal programs differ across regions, but a common thread is the active involvement of various stakeholders. In Thailand, although teachers may lack formal nutrition education, they are tasked with designing lunch menus and play a central role in monitoring students’ eating habits and encouraging healthier choices [82]. PNAE demonstrates the value of civil society partnerships, where non-profit organizations support female farmers by promoting agroecological farming techniques and guiding them through the organic certification process—efforts that contribute to greater food security and community empowerment [34]. Meanwhile, in the U.S., collaboration spans multiple levels, as federal agencies like the U.S.DA, local governments, and industry groups coordinate through initiatives such as Team Nutrition to provide training, financial resources, and advocacy that strengthen the overall quality of school meal programs [83].

Parental and community engagement plays a vital role in strengthening school meal programs across diverse settings. In several African countries, such as Kenya, Mauritania, Niger, and Sierra Leone, families and local communities support school feeding by contributing food, labor, and financial resources. In Guatemala and Liberia, parent organizations are actively involved in managing food procurement and meal distribution. School catering activities in Switzerland also include parental participation, reflecting a collaborative model of school meal management [43]. Thailand provides another example, where parents and caregivers regularly donate food and ingredients, highlighting how community contributions can enhance the success and sustainability of school meals [84]. In New Jersey, a study revealed that parental support played a significant role in sustaining student participation, even after the implementation of HHFKA [85]. Bangladesh’s program also emphasizes community mobilization, embedding local engagement into both implementation and monitoring processes [43].

The design and implementation model of school feeding programs can significantly impact their effectiveness. In Indonesia, a comparative study revealed that schools equipped with on-site kitchens and nutritionists delivered meals with better nutritional quality, hygiene, and portion control, while schools relying on off-site catering were more likely to face issues such as nutrient deficiencies and higher obesity rates among students [86]. In the U.S., initiatives like the Strong4Life training program in Georgia have enhanced cafeteria operations by improving staff knowledge and encouraging the use of healthier food presentation techniques and evidence-based practices [87]. South Korea’s experience highlights the importance of student satisfaction, which was strongly influenced by factors such as food quality, dining environment, and the quality of service management—demonstrating the value of a comprehensive, student-centered approach to school meals [88]. Local leadership can also make a difference, as seen in Tennessee, where school administrators improved meal quality by introducing new food policies and working with better service providers [89]. Additionally, the physical environment must not be overlooked. In Scotland, for instance, overcrowded dining halls were found to negatively affect participation in free school meal programs, underlining the importance of adequate infrastructure to support student engagement [90].

Effective strategies for enhancing school meal programs often focus on improving the nutritional quality of food offered. Studies have shown that increasing the availability of fruits and vegetables in school meals can lead to higher consumption among students [91]. Healthier meal options, even if they slightly reduce overall calorie intake, can significantly decrease fat and sodium levels [92]. Behavioral nudging techniques, such as encouraging students to choose healthier options like white milk through simple prompts, have also proven effective [93]. Further, strategic cafeteria interventions, such as marketing healthier food choices and removing unhealthy competitive foods, have increased meal participation in U.S. schools [94]. Programs that introduce salad bars, culturally relevant food options, and farm visits have also led to a significant rise in vegetable consumption among students [95,96]. Other studies also show that providing both healthy and less nutritious meals side-by-side often leads students to choose the latter, undermining the effectiveness of improved meal standards [97].

Government policies and regulations play a crucial role in shaping the quality and effectiveness of school meal programs. In the UK, the School Food Plan introduced key reforms, including mandatory cooking and nutrition education for younger students and stricter food standards, which collectively improved the quality of school meals [98]. In the U.S., the HHFKA brought significant changes to federal nutrition guidelines. However, some standards were influenced by industry lobbying. For example, allowing starchy vegetables and pizza sauce to count as part of a balanced meal [71]. Beyond nutrition standards, governments have also explored strategies to reduce food waste. Research shows that involving students in meal planning, scheduling longer lunch periods, and serving sliced fruits can lead to higher food consumption and less waste [74,99]. The timing of lunch and recess also matters: studies indicate that when recess is scheduled before lunch, children are more likely to eat fruits [99].

Collaboration and communication among stakeholders enhance the effectiveness of school meal programs. The UK’s HAF program benefited from strong partnerships between coordinators, providers, and parents, which improved communication, attendance, and access to meals during school holidays [19]. Similarly, innovations during the COVID-19 pandemic, such as regulatory waivers that allowed flexible meal distribution models, helped reduce barriers to participation in school meal programs [100]. Interviews with nutrition directors in North Carolina highlighted key factors influencing program implementation, such as leadership engagement, resource availability, and policy support, emphasizing the resilience of school meal programs in adapting to changing circumstances [101]. Additionally, social media campaigns played a role in advocating for the continuity of FSM [18].

#### 3.2.7. Barriers to a Successful School Lunch Program

School meal programs are critical in promoting child nutrition, supporting educational achievement, and alleviating food insecurity. However, despite their benefits, numerous implementation barriers persist across both high- and low-income countries. These barriers range from issues of food acceptance and meal quality to financial constraints and institutional inefficiencies, often varying by context yet sharing similar underlying patterns.

A major and recurring challenge lies in food acceptance among students. Even when healthy meals are provided, they are often rejected due to poor taste, unappealing presentation, or unfamiliar ingredients. In Thailand, for example, significant plate waste was reported, with students discarding large portions of rice, vegetables, and protein sources, undermining their overall nutrient intake [102]. Similarly, in China, students—especially younger ones—frequently wasted vegetables due to oversized portions and poor taste [103]. In the U.S., studies in Texas and South Carolina revealed that low-income or minority students consumed fewer calories or wasted more vegetables, especially those unfamiliar to them, such as dark green or red/orange varieties [92,104,105,106]. These findings highlight the need for meal customization that accounts for local taste preferences and age-appropriate portioning.

In addition to food acceptance, the overall nutritional quality of school meals remains inconsistent. While programs in countries like Japan and South Korea are known for structured, nutrient-dense offerings [107], other countries face issues related to overreliance on specific food groups. For instance, in Thailand, school lunches included excessive rice but lacked adequate portions of fruits, vegetables, and protein [102]. In China, imbalanced menus contributed to deficiencies in essential nutrients such as calcium, iron, and vitamin B2 [103]. Meanwhile, in the U.S., competitive foods often exceed sugar limits, reducing the overall nutritional value of school meals [108]. These examples reflect the tension between meeting caloric targets and ensuring micronutrient adequacy.

Financial limitations also significantly hinder program effectiveness, particularly in low- and middle-income settings. In Africa, only 38% of nations provide adequate funding for school meal programs, with challenges such as seasonal access and conflict exacerbating delivery issues [23]. In Kyrgyzstan and Thailand, delayed payments and budget constraints disrupt implementation [27,84], while in Ghana, inflation forces caterers to compromise menu quality [109]. Even in higher-income countries like the UK, the HAF Programme experienced a 16% funding decline since 2014, limiting meal variety and reducing sustainability [19,69]. In Canada, the absence of a federally funded program reflects a broader ideological stance that positions child nutrition as a family rather than state responsibility [110], creating a systemic gap in national food provision.

Beyond funding, logistical and infrastructural challenges also limit access and consistency. In several African regions, poor road conditions, weather, and displacement interfere with food delivery [23]. In the U.S., limited transportation and restricted meal-site hours reduced participation in summer feeding programs [100]. Additionally, FTS initiatives in Indiana struggled with unreliable supply chains and high costs of local produce, despite policy support [14,111]. These barriers suggest that supply chain reliability and local access must be addressed alongside nutritional goals.

Institutional coordination and program oversight further affect implementation. In the UK’s HAF programme, delayed contract awarding weakened nutrition education delivery and undermined overall program quality [19]. In Thailand, weak supervision and the absence of long-term monitoring systems were identified as critical bottlenecks, with school staff calling for greater support from external agencies [84]. Evidence from the U.S. also showed that school food departments with structured oversight were more successful in maintaining nutrition standards compared to schools where responsibilities were dispersed [108]. These cases illustrate the importance of governance structure, accountability mechanisms, and timely administrative processes.

Finally, human resource limitations and stakeholder engagement shape the feasibility of sustained school meal implementation. In North Carolina, cafeteria staff were overwhelmed with multitasking, leading to uneven program delivery [112]. Similarly, Dutch educators—while supportive in principle—raised concerns about increased workloads and questioned whether food provision should fall under school responsibilities [113]. Engaging teachers, parents, and students in program design can improve ownership and practicality while ensuring responsiveness to local needs.

### 3.3. Implication for Indonesian School Lunch Program: Redefining Purposes

The Indonesian government faces ongoing issues to overcome childhood malnutrition alongside food insecurity, while also providing balanced food to school children who live in less accessible areas. The distribution of essential nutrients in the population directly relates to structural problems, including social poverty and geographical barriers, and insufficient public health resources. The government introduces MBG as a national initiative program to supply free nutritious school meals throughout the country while setting up an IDR 71 trillion budget for 2025. This program offered an answer to dietary requirements and was a key component of a widespread social and political strategy that focused on economics. This study relies on policy evaluations and data from the Center for Indonesia’s Strategic Development Initiatives (CISDI), and studies from numerous nations, including Europe, America, Asia, and African countries that serve as international examples in this paper because they have a similar program to MBG.

#### 3.3.1. Redefining the Purpose of School Lunch Program for Indonesia

**Figure 5:**
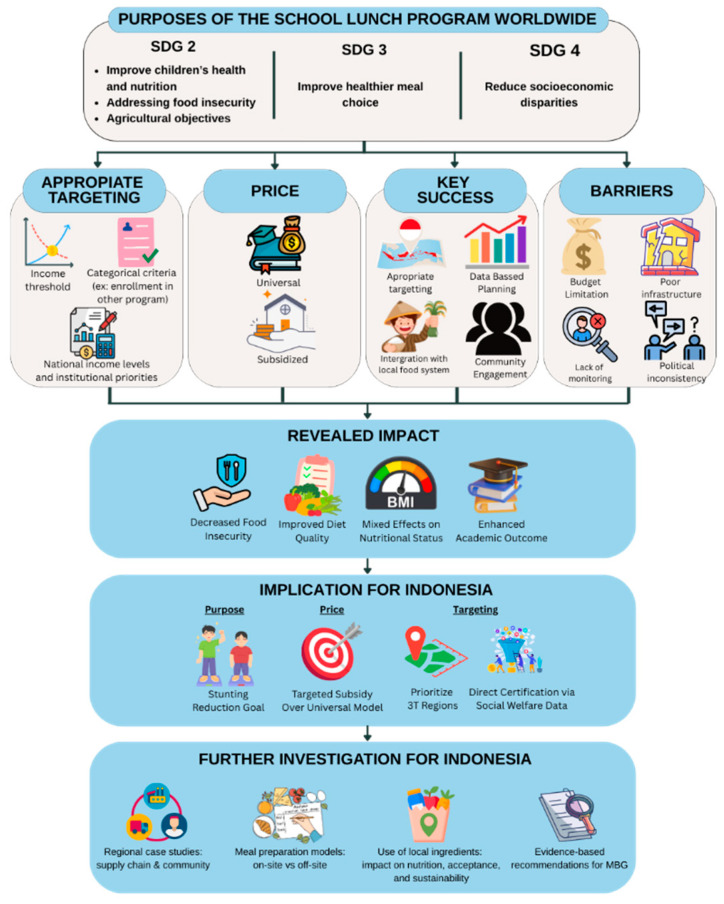
Summary of the insights.

The Prabowo–Gibran administration has launched the MBG program with the stated goal of reducing stunting in Indonesia. By 2025, the program aims to provide free nutritious meals to all Indonesian children, including specific interventions such as free meals for students and pregnant women, as well as free milk for school-aged children. While the program aspires to be universal (Figure 5), its initial focus is on children under six and students from low-income families in Indonesia’s most disadvantaged “3T” districts (Tertinggal, Terdepan, Terluar). Additional target groups include pregnant women, individuals with disabilities, and the elderly [114]. However, aligning the program’s purpose with the appropriate target population is crucial for achieving meaningful impact. According to the national stunting reduction strategy, *Percepatan Penurunan Stunting* (PPS), interventions should prioritize the first 1000 days of life—targeting pregnant and lactating women, children under five, and adolescent girls [114]. In Africa, China, and Kyrgyzstan, for instance, school meal programs are tailored specifically to rural or food-insecure areas, showing how geographic targeting can help address basic nutrition problems [23,26,27]. Expanding the MBG program beyond its early-life focus without clear differentiation risks diluting its impact on stunting outcomes.

A recent report by CISDI also highlights that MBG may not be the most strategic solution for stunting [114]. While the program may temporarily improve dietary intake and food access, it does not address the deeper root causes—such as poverty. To reduce socioeconomic disparities, countries like the U.S. and several in Africa focus their meal programs on low-income or vulnerable children, aiming to ease financial burdens and reduce stigma [23,33]. In Brazil and parts of Africa, meals are also linked to community agriculture through procurement from smallholder farmers—demonstrating how school feeding can support local economies while improving nutrition [14,29,34]. Meanwhile, South Korea and the UK combine school meals with nutrition education to instill healthy eating habits early on [31,32].

In this regard, programs like *Program Keluarga Harapan* (PKH), which provide targeted social protection to vulnerable families, may be more effective in addressing the socio-economic dimensions of stunting [114]. To ensure effectiveness, the MBG program must have a clearly defined purpose and target population. If the goal is to reduce stunting, the focus should remain on early life interventions. Conversely, if the aim is to improve general nutritional status or food security among school-aged children, then program design and resource allocation should reflect that shift.

#### 3.3.2. Cost Models in the MBG Program: Universal vs. Subsidized

There are mounting worries about money efficiency and vague goals of the IDR 71 trillion budget for Indonesia’s MBG scheme in 2025. Recent studies indicate that the universal method may amount to a waste of public monies spent on generalists and put financial stress on the government. Just like in the case of the UK’s Universal Infant Free School Meals (UIFSM), studies show that the universal school meal programs lead to greater consumption of nutrition [15,17,36]. However, with Indonesia’s fiscal challenges, the need for an evidence-based, well-planned approach arises.

Limited budget countries have discovered that subsidized models are more efficient. The USDA’s CNPs in the United States depend on income, while China’s NIPRCES pays schools directly to make local decisions. In Africa, partnerships between government, donors, and communities have sustained school feeding programs [23,26,35].

Both strategies have advantages, but Indonesia needs a model that is prepared to address its developmental and financial constraints. Inclusive approaches broadly contact constituents and reduce stigma, whereas subsidies provide concentrated advantages that optimize the usage of limited funds. In conclusion, it is recommended that a phased, partial-subsidy model, with emphasis on high-need areas and a performance evaluation method for subsequent growth, should be used. Such a strategy enhances participant involvement and program effectiveness as a whole and protects the integrity of central health and social services. For MBG to generate enduring value, its metrics should go beyond accounting for meals and shift to focus on improvements in child health equity and academic impact alongside the robustness of local food systems.

#### 3.3.3. Targeting Beneficiaries in the MBG Program: Equity and Justice Perspective

School lunch programs need proper targeting to successfully serve the individual who needs help the most. To reach vulnerable populations in challenging areas first, while laying the groundwork for a national rollout, we recommend a partial subsidy MBG model that combines geographic targeting with a CEP-style mechanism. Geographic targeting directs limited fiscal resources to Indonesia’s most disadvantaged “3T” districts (Tertinggal, Terdepan, Terluar). Meanwhile, a CEP-style approach uses existing social welfare data to automatically certify schools in high need. This aims to reduce paperwork, avoid stigma for low-income students, and minimize the risk of resources being misallocated or failing to reach the intended beneficiaries. Together, these strategies balance equity, efficiency, and administrative simplicity.

Prioritizing subsidies in 3T areas should be done as a primary step, because these areas consistently show the highest levels of malnutrition among school-age children [115]. In addition, the government already has an official list of 3T districts and sub-districts, which is stated in Presidential Regulation (Perpres) Number 63 of 2020 on Determination of Underdeveloped Regions in 2020–2024. This aims to eliminate the delay and cost of new surveys or mean-testing at each school. By channeling subsidies where they are needed most and with the data infrastructure already in place, MBG can maximize impact per rupiah spent.

However, relying solely on geographic targeting risks excluding vulnerable children outside designated 3T areas, such as those in urban slums or suburban communities, who may experience similar levels of malnutrition. To address this, the MBG program could adopt a CEP-style direct-certification model, similar to that used in the U.S. [40]. Under this model, schools with a significant proportion of “identified students”—children already enrolled in social-protection schemes like Penerima Bantuan Iuran Jaminan Kesehatan Nasional (PBI-JKN) or Program Keluarga Harapan (PKH)—automatically qualify for higher subsidy tiers without requiring additional household applications.

Specifically, MBG could set an Identified Student Percentage (ISP) threshold—like the U.S. CEP’s ≥ 40 percent rule—above which a school receives universal free meals funded centrally, while those just below the threshold receive a scaled partial subsidy. This approach promotes both equity and efficiency. Over time, as data coverage and budgets improve, MBG can lower the ISP threshold and extend the model beyond 3T regions, ensuring a clear, data-driven pathway to nationwide expansion.

## 4. Conclusions

The study’s bibliometric analysis was crucial in identifying key research trends and gaps in global school lunch programs, providing a valuable foundation for the proposed framework for Indonesia’s MBG. The analysis revealed an increasing focus on sustainability, food waste management, and the integration of nutrition education into school curricula. It also highlighted significant disparities in implementation and accessibility, particularly in low-income regions. The most prominent research themes identified were related to government-backed programs like the “national school lunch program” and their impact on child health and food security, as well as the socioeconomic dimensions of these programs, including their role in addressing food insecurity among low-income households. Emerging trends included the impact of the COVID-19 pandemic on school nutrition programs and a growing interest in policies that ensure equitable meal access for all students. Overall, the MBG program should concentrate on a system where subsidies are offered gradually and only to high-need districts. The program should be tailored to support early efforts that will reduce stunting. Implementing geographic targeting in 3T regions along with a CEP system can improve how fair and simple the system is. By using available social welfare data and deciding on clear limits, MBG should reduce the risk of leaving anyone behind and avoid wasting resources, laying a good foundation for future expansion. Measuring success by the effects on health and education is better than counting meals served alone.

This study, however, has limitations. While it offers a broad global review, the generalizability of its findings to Indonesia may be constrained by local specificities such as cultural and gastronomic preferences, local topography, and heritage. For instance, what works in a universal program in South Korea or a targeted one in rural China may not be directly applicable to Indonesia’s diverse archipelagic context. Future research should address these gaps by focusing on case studies that explore the effectiveness of school feeding programs within Indonesia’s unique regional contexts, considering the challenges of supply chain reliability and community engagement in diverse geographical settings. Investigating how different meal preparation models (on-site versus off-site catering) and the integration of local ingredients affect nutritional quality, student acceptance, and economic sustainability in various Indonesian regions would also be beneficial. These future studies could provide more tailored, evidence-based recommendations to optimize the MBG program and ensure its long-term success.

## Figures and Tables

**Table 1:** Key Goals of School Lunch Program Globally.

Purpose	Country
Improve children’s health and nutrition	Africa, China, Kyrgyzstan
Improve healthier meal choices	UK, South Korea
Reduce socioeconomic disparities	Africa, U.S.
Agricultural objectives	Africa, Brazil
Addressing food insecurity	U.S.

## Data Availability

The original contributions presented in the study are included in the article, further inquiries can be directed to the corresponding author/s.

## References

[1] Naspolini N.F., Schüroff P.A., Vanzele P.A.R., Pereira-Santos D., Valim T.A., Bonham K.S., Fujita A., Passos-Bueno M.R., Beltrão-Braga P.C.B., Carvalho A.C.P.L.F. (2025). Exclusive Breastfeeding Is Associated with the Gut Microbiome Maturation in Infants According to Delivery Mode. Gut Microbes.

[2] Joint Child Malnutrition Estimates. https://www.who.int/data/gho/data/themes/topics/joint-child-malnutrition-estimates-unicef-who-wb.

[3] Ashar H., Laksono A.D., Supadmi S., Kusumawardani H.D., Yunitawati D., Purwoko S., Khairunnisa M. (2024). Factors Related to Stunting in Children under 2 Years Old in the Papua, Indonesia: Does the Type of Residence Matter?. Saudi Med. J..

[4] Cendana P., Kim S.-Y. (2025). Maternal Factors and Breastfeeding Practices Associated with Stunting among Indonesian Children Aged 6 to 23 Months. Asia Pac. J. Public Health.

[5] Yantri N.F., Simbolon D., Agustina D., Syaefatul D., Firnawati F., Hertipa F., Febryanti F., Putri O.J. (2025). The Proportion of Animal Protein Consumption with A Prevalence of Stunting, Wasting, and Underweight in Toddlers in Indonesia. Proceeding Int. Conf. Health Polytech. Jambi.

[6] Azriani D., Masita, Qinthara N.S., Yulita I.N., Agustian D., Zuhairini Y., Dhamayanti M. (2024). Risk Factors Associated with Stunting Incidence in under Five Children in Southeast Asia: A Scoping Review. J. Health Popul. Nutr..

[7] De Sanctis V., Soliman A., Alaaraj N., Ahmed S., Alyafei F., Hamed N. (2021). Early and Long-Term Consequences of Nutritional Stunting: From Childhood to Adulthood. Acta Biomed..

[8] Bundy D., Gentilini U., Schultz L., Bedasso B., Singh S., Okamura Y., Iyengar H.T., Blakstad M. (2024). School Meals, Social Protection, and Human Development: Revisiting Trends, Evidence, and Practices in South Asia and Beyond.

[9] An Agenda to Combat Child Hunger; Strengthen Equity School Feeding and the Sustainable Development Goals. https://schoolmealscoalition.org/sites/default/files/2024-11/ODI_School_feeding_and_the_SDGs.pdf.

[10] Open Knowledge Repository. https://openknowledge.worldbank.org/bitstreams/66305c9b-a4c8-52c2-89c9-19d1b219862d/download.

[11] Busey E.A., Chamberlin G., Mardin K., Perry M., Taillie L.S., Dillman Carpentier F.R., Popkin B.M. (2024). National Policies to Limit Nutrients, Ingredients, or Categories of Concern in School Meals: A Global Scoping Review. Curr. Dev. Nutr..

[12] Donthu N., Kumar S., Mukherjee D., Pandey N., Lim W.M. (2021). How to Conduct a Bibliometric Analysis: An Overview and Guidelines. J. Bus. Res..

[13] Singh V.K., Singh P., Karmakar M., Leta J., Mayr P. (2021). The Journal Coverage of Web of Science, Scopus and Dimensions: A Comparative Analysis. Scientometrics.

[14] Gibson C.A., Harvey S.P., Spaeth K., Sullivan D.K., Lambourne K., Kunkel G.H. (2014). Farm to School, School to Home: An Evaluation of a Farm to School Program at an Urban Core Head Start Preschool Program. J. Hunger Environ. Nutr..

[15] Yang T.C., Power M., Moss R.H., Lockyer B., Burton W., Doherty B., Bryant M. (2022). Are Free School Meals Failing Families? Exploring the Relationship between Child Food Insecurity, Child Mental Health and Free School Meal Status during COVID-19: National Cross-Sectional Surveys. BMJ Open.

[16] Taylor C. (2018). The Reliability of Free School Meal Eligibility as a Measure of Socio-Economic Disadvantage: Evidence from the Millennium Cohort Study in Wales. Br. J. Educ. Stud..

[17] Goodchild G.A., Faulks J., Swift J.A., Mhesuria J., Jethwa P., Pearce J. (2017). Factors Associated with Universal Infant Free School Meal Take up and Refusal in a Multicultural Urban Community. J. Hum. Nutr. Diet..

[18] Parnham J.C., McKevitt S., Vamos E.P., Laverty A.A. (2023). Evidence Use in the UK’s COVID-19 Free School Meals Policy: A Thematic Content Analysis. Policy Des. Pract..

[19] Stringer A., Bayes N., Bradley S., Kay A.D., Jones P.G.W., Ryan D.J. (2022). A Mixed-Method Process Evaluation of an East Midlands County Summer 2021 Holiday Activities and Food Programme Highlighting the Views of Programme Co-Ordinators, Providers, and Parents. Front. Public Health.

[20] Guio A.-C. (2023). Free School Meals for All Poor Children in Europe: An Important and Affordable Target?. Child. Soc..

[21] Ray C., Roos E., Brug J., Behrendt I., Ehrenblad B., Yngve A., te Velde S.J. (2013). Role of Free School Lunch in the Associations between Family-Environmental Factors and Children’s Fruit and Vegetable Intake in Four European Countries. Public Health Nutr..

[22] Vik F.N., Van Lippevelde W., Øverby N.C. (2019). Free School Meals as an Approach to Reduce Health Inequalities among 10-12- Year-Old Norwegian Children. BMC Public Health.

[23] Wineman A., Ekwueme M.C., Bigayimpunzi L., Martin-Daihirou A., de Gois V N Rodrigues E.L., Etuge P., Warner Y., Kessler H., Mitchell A. (2022). School Meal Programs in Africa: Regional Results from the 2019 Global Survey of School Meal Programs. Front. Public Health.

[24] Kim M., Abe S., Zhang C., Kim S., Choi J., Hernandez E., Nozue M., Yoon J. (2017). Comparison of the Nutrient-Based Standards for School Lunches among South Korea, Japan, and Taiwan. Asia Pac. J. Clin. Nutr..

[25] Baek D., Choi Y., Lee H. (2019). Universal Welfare May Be Costly: Evidence from School Meal Programs and Student Fitness in South Korea. Sustainability.

[26] Wang J., Hernandez M.A., Deng G. (2023). Large-Scale School Meal Programs and Student Health: Evidence from Rural China. China Econ. Rev..

[27] Kyzy A.M. (2019). School Attendance: Demographic Differences and the Effect of a Primary School Meal Programme in Kyrgyzstan. Educ. Res. Eval..

[28] Borlizzi A., Delgrossi M.E., Cafiero C. (2017). National Food Security Assessment through the Analysis of Food Consumption Data from Household Consumption and Expenditure Surveys: The Case of Brazil’s Pesquisa de Orçamento Familiares 2008/09. Food Policy.

[29] De Carvalho Souza Machado T., Cristina Apolinario Borges C., Coelho Ribeiro Mendonca F., Cristina Euzebio Pereira Dias de Oliveira B. (2020). Parasitological Evaluation of Lettuce Served in School Meals at a Federal State School in Rio DE Janeiro, Brazil. Rev. Patol. Trop..

[30] Rocha C. (2009). Developments in National Policies for Food and Nutrition Security in Brazil. Dev. Policy Rev..

[31] Lee Y., Kim O., Lee U., Kwon S. (2017). Evaluation of Educational School Meal Programs in Gyeonggi Province, South Korea. J. Nutr. Health.

[32] Elliott V., Hore B. (2016). ‘Right Nutrition, Right Values’: The Construction of Food, Youth and Morality in the UK Government 2010–2014. Camb. J. Educ..

[33] Chapman L.E., Gosliner W., Olarte D.A., Ritchie L.D., Schwartz M.B., Polacsek M., Hecht C.E., Hecht K., Turner L., Patel A.I. (2024). Universal School Meals during the Pandemic: A Mixed Methods Analysis of Parent Perceptions from California and Maine. J. Acad. Nutr. Diet..

[34] Valencia V., Wittman H., Jones A.D., Blesh J. (2021). Public Policies for Agricultural Diversification: Implications for Gender Equity. Front. Sustain. Food Syst..

[35] Zafari Z., Cohen J.F.W., Sessom-Parks L., Lessard D., Cooper M., Hager E. (2023). Impacts of COVID-19 School Closures on School Food Service Revenue: Analysis of Public Local Education Agencies in Maryland. J. Sch. Health.

[36] Peterson C., Le Grand J. (2011). Should the U.S. National School Lunch Program Continue Its In-kind Food Benefit? A School District-level Analysis of Funding Efficiency and Equity. Appl. Econ. Perspect. Policy.

[37] Milne R.G., Gibb K. (2016). Using Economic Analysis to Increase Civic Engagement. Contemp. Soc. Sci..

[38] Kim J., Joo M. (2020). The Effects of Direct Certification in the US National School Lunch Program on Program Participation. J. Soc. Soc. Work. Res..

[39] Huang J., Kim Y., Barnidge E. (2016). Seasonal Difference in National School Lunch Program Participation and Its Impacts on Household Food Security. Health Soc. Work.

[40] Kashyap P., Jablonski B.B.R. (2024). Universal Free School Meals: Examining Factors Influencing Adoption of the Community Eligibility Provision. Appl. Econ. Perspect. Policy.

[41] Rosen R., Dickson E. (2024). The Exceptions to Child Exceptionalism: Racialised Migrant ‘Deservingness’ and the UK’s Free School Meal Debates. Crit. Soc. Policy.

[42] Food and Agriculture Organization of the United Nations (2019). Nutrition Guidelines and Standards for School Meals: A Report from 33 Low and Middle-Income Countries.

[43] Global Child Nutrition Foundation (2019). School Meal Programs Around the World: Report Based on the Global Survey of School Meal Programs.

[44] Dall’ACqua F.M. (1991). Economic Adjustment and Nutrition Policies: Evaluation of a School-Lunch Programme in Brazil. Food Nutr. Bull..

[45] Spill M.K., Trivedi R., Thoerig R.C., Balalian A.A., Schwartz M.B., Gundersen C., Odoms-Young A., Racine E.F., Foster M.J., Davis J.S. (2024). Universal Free School Meals and School and Student Outcomes: A Systematic Review. JAMA Netw. Open.

[46] Gundersen C., Ziliak J.P. (2018). Food Insecurity Research in the United States: Where We Have Been and Where We Need to Go. Appl. Econ. Perspect. Policy.

[47] Arteaga I., Heflin C. (2014). Participation in the National School Lunch Program and Food Security: An Analysis of Transitions into Kindergarten. Child. Youth Serv. Rev..

[48] Smith T.A. (2017). Do School Food Programs Improve Child Dietary Quality?. Am. J. Agric. Econ..

[49] Forrestal S., Potamites E., Guthrie J., Paxton N. (2021). Associations among Food Security, School Meal Participation, and Students’ Diet Quality in the First School Nutrition and Meal Cost Study. Nutrients.

[50] Kuhn M.A. (2018). Who Feels the Calorie Crunch and When? The Impact of School Meals on Cyclical Food Insecurity. J. Public Econ..

[51] Huang J., Barnidge E., Kim Y. (2015). Children Receiving Free or Reduced-Price School Lunch Have Higher Food Insufficiency Rates in Summer. J. Nutr..

[52] Vik F.N., Næss I.K., Heslien K.E.P., Øverby N.C. (2019). Possible Effects of a Free, Healthy School Meal on Overall Meal Frequency among 10-12-Year-Olds in Norway: The School Meal Project. BMC Res. Notes.

[53] Carlisle V.R., Jessiman P.E., Breheny K., Campbell R., Jago R., Leonard N., Robinson M., Strong S., Kidger J. (2023). A Mixed Methods, Quasi-Experimental Evaluation Exploring the Impact of a Secondary School Universal Free School Meals Intervention Pilot. Int. J. Environ. Res. Public Health.

[54] Mirtcheva D.M., Powell L.M. (2013). National School Lunch Program Participation and Child Body Weight. East. Econ. J..

[55] Capogrossi K., You W. (2017). The Influence of School Nutrition Programs on the Weight of Low-Income Children: A Treatment Effect Analysis. Health Econ..

[56] Li J., Hooker N.H. (2010). Childhood Obesity and Schools: Evidence from the National Survey of Children’s Health. J. Sch. Health.

[57] Bardin S., Gola A.A. (2020). Analyzing the Association between Student Weight Status and School Meal Participation: Evidence from the School Nutrition and Meal Cost Study. Nutrients.

[58] Localio A.M., Knox M.A., Basu A., Lindman T., Walkinshaw L.P., Jones-Smith J.C. (2024). Universal Free School Meals Policy and Childhood Obesity. Pediatrics.

[59] Holford A., Rabe B. (2024). Universal Free School Meals and Children’s Bodyweight. Impacts by Age and Duration of Exposure. J. Health Econ..

[60] Bethmann D., Cho J.I. (2022). The Impacts of Free School Lunch Policies on Adolescent BMI and Mental Health: Evidence from a Natural Experiment in South Korea. SSM Popul. Health.

[61] Chang H.-H. (2014). Food Preparation for the School Lunch Program and Body Weight of Elementary School Children in Taiwan. Int. Food Agribus. Manag. Rev..

[62] Figlio D.N., Winicki J. (2005). Food for Thought: The Effects of School Accountability Plans on School Nutrition. J. Public Econ..

[63] Thompson E.D. (2020). Why Nutritious Meals Matter in School. Phi Delta Kappan.

[64] Adrogue C., Orlicki M.E. (2013). Do In-School Feeding Programs Have an Impact on Academic Performance and Dropouts? The Case of Public Schools in Argentina. Educ. Policy Anal. Arch..

[65] Armitage E., Lau C. (2020). Can the English Baccalaureate Act as an Educational Equaliser?. Assess Educ..

[66] Cullen K.W., Chen T.-A. (2017). The Contribution of the USDA School Breakfast and Lunch Program Meals to Student Daily Dietary Intake. Prev. Med. Rep..

[67] Patel K., Strait K., Hildebrand D., Amaya L., Joyce J. (2020). Variability in Dietary Quality of an Elementary School Lunch Menu with Changes in National School Lunch Program Nutrition Standards. Curr. Dev. Nutr..

[68] Day R.E., Sahota P., Christian M.S., Cocks K. (2015). A Qualitative Study Exploring Pupil and School Staff Perceptions of School Meal Provision in England. Br. J. Nutr..

[69] Parnham J.C., Chang K., Rauber F., Levy R.B., Laverty A.A., Pearson-Stuttard J., White M., von Hinke S., Millett C., Vamos E.P. (2024). Evaluating the Impact of the Universal Infant Free School Meal Policy on the Ultra-Processed Food Content of Children’s Lunches in England and Scotland: A Natural Experiment. Int. J. Behav. Nutr. Phys. Act..

[70] Gu X., Tucker K.L. (2017). Dietary Quality of the US Child and Adolescent Population: Trends from 1999 to 2012 and Associations with the Use of Federal Nutrition Assistance Programs. Am. J. Clin. Nutr..

[71] Cohen J., Schwartz M.B. (2020). Documented Success and Future Potential of the Healthy, Hunger-Free Kids Act. J. Acad. Nutr. Diet..

[72] Berger A.T., Widome R., Erickson D.J., Laska M.N., Harnack L.J. (2020). Changes in Association between School Foods and Child and Adolescent Dietary Quality during Implementation of the Healthy, Hunger-Free Kids Act of 2010. Ann. Epidemiol..

[73] Terry-McElrath Y.M., O’Malley P.M., Johnston L.D. (2015). Foods and Beverages Offered in US Public Secondary Schools through the National School Lunch Program from 2011–2013: Early Evidence of Improved Nutrition and Reduced Disparities. Prev. Med..

[74] Buscemi J., Odoms-Young A., Yaroch A.L., Hayman L.L., Loiacono B., Herman A., Fitzgibbon M.L. (2019). Society of Behavioral Medicine Position Statement: Retain School Meal Standards and Healthy School Lunches. Transl. Behav. Med..

[75] Meng Y., Manore M.M., Schuna J.M., Patton-Lopez M.M., Branscum A., Wong S.S. (2018). Promoting Healthy Diet, Physical Activity, and Life-Skills in High School Athletes: Results from the WAVE Ripples for Change Childhood Obesity Prevention Two-Year Intervention. Nutrients.

[76] Gearan E.C., Monzella K., Gola A.A., Figueroa H. (2021). Adolescent Participants in the School Lunch Program Consume More Nutritious Lunches but Their 24-Hour Diets Are Similar to Nonparticipants. J. Adolesc. Health.

[77] Mansfield J.L., Savaiano D.A. (2017). Effect of School Wellness Policies and the Healthy, Hunger-Free Kids Act on Food-Consumption Behaviors of Students, 2006-2016: A Systematic Review. Nutr. Rev..

[78] Bean M.K., Brady Spalding B., Theriault E., Dransfield K.-B., Sova A., Dunne Stewart M. (2018). Salad Bars Increased Selection and Decreased Consumption of Fruits and Vegetables 1 Month after Installation in Title I Elementary Schools: A Plate Waste Study. J. Nutr. Educ. Behav..

[79] Cullen K.W., Watson K., Zakeri I., Ralston K. (2006). Exploring Changes in Middle-School Student Lunch Consumption after Local School Food Service Policy Modifications. Public Health Nutr..

[80] Wu S., Yoder G., Lee N.Y., Yan S., Wolfersteig W. (2021). Racial Disparities in School Lunch Program Participation and Cigarette Use: Evidence from Arizona Youth Survey Data. Subst. Use Misuse.

[81] Fox M.K., Gearan E.C., Schwartz C. (2021). Added Sugars in School Meals and the Diets of School-Age Children. Nutrients.

[82] Keeratichamroen A., Praditsorn P., Churak P., Srisangwan N., Sranacharoenpong I., Ponprachanuvut P., Chammari A., Sranacharoenpong K., Chammari K. (2024). Applying the Concept of Thai Nutrient Profiling as a Model for the Thai School Lunch Planner. J. Public Health Dev..

[83] Schultz C., Thorlton J. (2019). Access to Fresh Fruits and Vegetables in School Lunches: A Policy Analysis. J. Sch. Nurs..

[84] Srisangwan N., Churak P., Praditsorn P., Ponprachanuvut P., Keeratichamroen A., Chammari K., Sranacharoenpong K. (2023). Using SWOT Analysis to Create Strategies for Solving Problems in Implementing School Lunch Programs in Thailand. J. Health Res..

[85] Martinelli S., Acciai F., Au L.E., Yedidia M.J., Ohri-Vachaspati P. (2020). Parental Perceptions of the Nutritional Quality of School Meals and Student Meal Participation: Before and after the Healthy Hunger-Free Kids Act. J. Nutr. Educ. Behav..

[86] Ronitawati P., Setiawan B., Sinaga T. (2020). The Influence of Nutritionist-Based Food Service Delivery System on Food and Nutrient Quality of School Lunch Program in Primary Schools in Indonesia. J. Nutr. Sci. Vitaminol..

[87] Rajbhandari-Thapa J., Bennett A., Keong F., Palmer W., Hardy T., Welsh J. (2017). Effect of the Strong4Life School Nutrition Program on Cafeterias and on Manager and Staff Member Knowledge and Practice, Georgia, 2015. Public Health Rep..

[88] Kwon S., Kim O., Lee Y. (2018). Effects of Students’ Satisfaction with School Meal Programs on School Happiness in South Korea. Nutr. Res. Pract..

[89] Trapp M.M. (2018). The Right to Taste: Conceptualizing the Nourishing Potential of School Lunch. Food Foodways.

[90] Chambers S., Dundas R., Torsney B. (2016). School and Local Authority Characteristics Associated with Take-up of Free School Meals in Scottish Secondary Schools, 2014. Contemp. Soc. Sci..

[91] Newman C. (2013). Fruit and Vegetable Consumption by School Lunch Participants: Implications for the Success of New Nutrition Standards.

[92] Peckham J.G., Kropp J.D., Mroz T.A., Haley-Zitlin V., Granberg E.M., Hawthorne N. (2017). Socioeconomic and Demographic Determinants of the Nutritional Content of National School Lunch Program Entrée Selections. Am. J. Agric. Econ..

[93] Lai C.-Y., List J.A., Samek A. (2020). Got Milk? Using Nudges to Reduce Consumption of Added Sugar. Am. J. Agric. Econ..

[94] Boehm R., Read M., Henderson K.E., Schwartz M.B. (2020). Removing Competitive Foods v. Nudging and Marketing School Meals: A Pilot Study in High-School Cafeterias. Public Health Nutr..

[95] Zhong A., Yin L., O’Sullivan B., Ruetz A.T. (2023). Historical Lessons for Canada’s Emerging National School Food Policy: An Opportunity to Improve Child Health. Health Promot. Chronic Dis. Prev. Can..

[96] Slusser W.M., Cumberland W.G., Browdy B.L., Lange L., Neumann C. (2007). A School Salad Bar Increases Frequency of Fruit and Vegetable Consumption among Children Living in Low-Income Households. Public Health Nutr..

[97] Joyce J.M., Harris K., Mailey E.L., Rosenkranz R.R., Rosenkranz S.K. (2020). Acceptability and Feasibility of Best Practice School Lunches by Elementary School-Aged Children in a Serve Setting: A Randomized Crossover Trial. Int. J. Environ. Res. Public Health.

[98] Schabas L. (2014). The School Food Plan: Putting Food at the Heart of the School Day. Nutr. Bull..

[99] Chapman L.E., Cohen J., Canterberry M., Carton T.W. (2017). Factors Associated with School Lunch Consumption: Reverse Recess and School “Brunch”. J. Acad. Nutr. Diet..

[100] Bennett B.L., McKee S.L., Burkholder K., Chafouleas S.M., Schwartz M.B. (2024). USDA’s Summer Meals during the COVID-19 Pandemic: A Mixed-Methods Examination of Participants and Non-Participants in 2021. J. Acad. Nutr. Diet..

[101] Katz B.N., Soldavini J., Grover K., Jilcott Pitts S., Martin S.L., Thayer L., Ammerman A.S., Lane H.G. (2022). “let’s Use This Mess to Our Advantage”: Calls to Action to Optimize School Nutrition Program beyond the Pandemic. Int. J. Environ. Res. Public Health.

[102] Petchoo J., Kaewchutima N., Tangsuphoom N. (2022). Nutritional Quality of Lunch Meals and Plate Waste in School Lunch Programme in Southern Thailand. J. Nutr. Sci..

[103] Huang Z., Gao R., Bawuerjiang N., Zhang Y., Huang X., Cai M. (2017). Food and Nutrients Intake in the School Lunch Program among School Children in Shanghai, China. Nutrients.

[104] Ishdorj A., Capps O., Murano P.S. (2016). Nutrient Density and the Cost of Vegetables from Elementary School Lunches. Adv. Nutr..

[105] Niaki S.F., Moore C.E., Chen T.-A., Weber Cullen K. (2017). Younger Elementary School Students Waste More School Lunch Foods than Older Elementary School Students. J. Acad. Nutr. Diet..

[106] Peckham J.G., Kropp J.D., Mroz T.A., Haley-Zitlin V., Granberg E.M. (2019). Selection and Consumption of Lunches by National School Lunch Program Participants. Appetite.

[107] Meier C.L., Brady P., Askelson N., Ryan G., Delger P., Scheidel C. (2022). What Do Parents Think about School Meals? An Exploratory Study of Rural Middle School Parents’ Perceptions. J. Sch. Nurs..

[108] Cohen J.F.W., Kesack A., Daly T.P., Elnakib S.A., Hager E., Hahn S., Hamlin D., Hill A., Lehmann A., Lurie P. (2024). Competitive Foods’ Nutritional Quality and Compliance with Smart Snacks Standards: An Analysis of a National Sample of U.s. Middle and High Schools. Nutrients.

[109] Liguori J., Amevinya G.S., Holdsworth M., Savy M., Laar A. (2024). Nutritional Quality and Diversity in Ghana’s School Feeding Programme: A Mixed-Methods Exploration through Caterer Interviews in the Greater Accra Region. BMC Nutr..

[110] Carbone S., Power E., Holland M.R. (2020). Canada’s Missed Opportunity to Implement Publicly Funded School Meal Programs in the 1940s. Crit. Public Health.

[111] Boling P., Blackburn E., Paine J., Smith R. (2018). Farm-to-School in Indiana: The Local Politics of Feeding Children. J. Hunger Environ. Nutr..

[112] Calancie L., Soldavini J., Dawson-McClure S. (2018). Partnering to Strengthen School Meals Programs in a Southeastern School District. Prog. Community Health Partnersh..

[113] van Kleef E., Dijkstra S.C., Seidell J., Vingerhoeds M.H., Polet I.A., Zeinstra G.G. (2022). Which Factors Promote and Prohibit Successful Implementation and Normalization of a Healthy School Lunch Program at Primary Schools in the Netherlands?. J. Health Popul. Nutr..

[114] Center for Indonesia’s Strategic Development Initiatives (2025). Mengkaji Ulang Program Makan Bergizi Gratis Makan Bergizi Gratis: Menilik Tujuan, Anggaran Dan Tata Kelola Program.

[115] Juliannisa I.A., Rahma H., Mulatsih S., Fauzi A. (2025). Regional Vulnerability to Food Insecurity: The Case of Indonesia. Sustainability.

